# HIV-Envelope–Dependent Cell-Cell Fusion: Quantitative Studies

**DOI:** 10.1100/tsw.2009.90

**Published:** 2009-08-11

**Authors:** Leonor Huerta, Nayali López-Balderas, Evelyn Rivera-Toledo, Guadalupe Sandoval, Guillermo Gómez-Icazbalceta, Carlos Villarreal, Edmundo Lamoyi, Carlos Larralde

**Affiliations:** ^1^ Departamento de Inmunología, Instituto de Investigaciones Biomédicas, Universidad Nacional Autónoma de México, Ciudad Universitaria, Distrito Federal, CP 04510, Mexico; ^2^ Departamento de Física Teórica, Instituto de Física, Universidad Nacional Autónoma de México, Ciudad Universitaria, Distrito Federal, CP 04510, Mexico

**Keywords:** cell-cell fusion, HIV, syncytia, syncytium-inducing virus, flow cytometry, Env, cell aggregation, FRET

## Abstract

Interaction *in vitro* between cells infected with human immunodeficiency virus (HIV) and surrounding, uninfected, target cells often leads to cell fusion and the formation of multinucleated cells, called syncytia. The presence in HIV-infected individuals of virus strains able to induce syncytia in cultures of T cells is associated with disease progression and AIDS. Even in the asymptomatic stage of infection, multinucleated cells have been observed in different organs, indicating that fused cells may be generated and remain viable in the tissues of patients. We used lymphocytic cells transfected for the expression of the HIV-envelope (Env) glycoproteins to develop a method for the direct quantification of fusion events by flow cytometry (Huerta et al., 2006, *J. Virol. Methods* 138, 17–23; López-Balderas et al., 2007, *Virus Res.* 123, 138–146). The method involves the staining of fusion partners with lipophilic probes and the use of fluorescence resonance energy transfer (FRET) to distinguish between fused and aggregated cells. We have shown that such a flow-cytometry assay is appropriate for the screening of compounds that have the potential to modulate HIV-Env–mediated cell fusion. Even those syncytia that are small or few in numbers can be detected. Quantitative analysis of the fusion products was performed with this technique; the results indicated that the time of reaction and initial proportion of fusion partners determine the number, relative size, and average cellular composition of syncytia. Heterogeneity of syncytia generated by HIV-Env–mediated cell-cell fusion may result in a variety of possible outcomes that, in turn, may influence the biological properties of the syncytia and surrounding cells, as well as replication of virus. Given the myriad immune abnormalities leading to AIDS, the full understanding of the extent, diverse composition, and role of fused cells in the pathogenesis of, and immune response to, HIV infection is an important, pending issue.

## CELL-CELL FUSION IN HIV INFECTION

Cell-cell fusion is a form of cell organization that occurs in physiological and pathological conditions, such as the formation of muscle[1] and placenta[2], development in metazoa[3], organ repair by stem cells[4,5,6,7], and malignant transformation[8,9,10]. Fusion of cells of the immune system is induced in several different infections and in noninfectious reactions to tissue injury[11,12]. This intimate association between cells from the same, or different, lineages (forming syncytia or heterokaryons, respectively) may result in a highly functional synchronization and an optimal efficiency in order to accomplish complex tasks, and may involve a major cellular reprogramming, resulting in an altered phenotype[8,13,14,15]. Thus, the properties of fused cells usually differ significantly from those of parental cells. An excellent review by Ogle et al.[10] describes the general biological implications of cell-cell fusion.

A variety of enveloped viruses, including the human immunodeficiency virus (HIV), harbor "spikes" composed of glycoproteins that interact with receptors on target cells, thus inducing virus-cell fusion. Infected cells synthesize and then expose the viral fusion proteins on their membranes, thereby conferring the infected cells with the ability to fuse with uninfected cells. Included among the viruses that induce cell-cell fusion are many viruses that are associated with human diseases, such as the Herpesviridae (herpes viruses, cytomegalovirus, varicella zoster, Epstein-Barr virus), Paramyxoviridae (measles, mumps, syncytial respiratory virus), and Retroviridae (VIH, HTLV) families. The ability of viruses to induce syncytia is generally assumed to be indicative of the viruses being transmitted through a cell-to-cell pathway, without exposure to the extracellular milieu, thus allowing virus to spread rapidly and to evade the immune response of the host[16]. However, the extent and consequences of syncytia formation during infection by these viruses are still poorly understood. Table 1 shows examples of specific pathogenic processes in viral infections with which syncytia formation has been associated**.** Given the relationship between several fusogenic viruses and cancer, Duelli and Lazebnik have considered the intriguing possibility that cell transformation arises from the combination of oncogene activity and chromosomal instability induced by cell-cell fusion[9].

Cell adhesion molecules[33,34,35,36] and fusion proteins[37,38] are used by viruses to overcome the electrostatic barrier formed by the negatively charged surface of cell membranes. Cell fusion requires the close apposition of the surface membranes of cells, the induction of lipid perturbations, and the formation of fusion pores that eventually expand and lead to complete cell-cell fusion[39]. During HIV infection, virus-membrane fusion is accomplished by the HIV-envelope (Env) macromolecular complex, composed of heterotrimers of the gp120 and gp41 glycoproteins. Fusion requires a sequence of complex conformational changes that involve the specific interaction of Env with the CD4 molecule and a coreceptor of the chemokine receptor family[40,41,42]. HIV-Env–mediated cell-cell fusion is induced by a mechanism analogous to that of the virus-cell fusion process[43,44,45].

In contrast to the advances in the understanding of the molecular processes of HIV-induced membrane fusion, the factors involved in the cell-cell fusion and the biological role of fused cells in HIV infection has not been well studied. In early investigations, syncytia formation was described as a form of cytopathic effect occurring when mononuclear cells from HIV-infected individuals were cocultured with uninfected T $CD4^+$ cells[43,44,45]. Later reports consistently showed that detection of virus with the ability to induce syncytia in cultures of T lymphocytes correlates with an increased rate of depletion of $CD4^+$ T lymphocytes and with progression to AIDS[46,47,48,49]. Multinucleated cells supporting virus replication were detected in different tissues, such as brain and lymphoid organs, of HIV-infected individuals, even when these patients were in the asymptomatic stage of the disease[50,51,52,53,54,55]. That syncytia may originate from the fusion of monocytes, lymphocytes, and/or dendritic cells has been evidenced by both the anatomical location and the phenotypic markers of the multinucleated cells[52,54,56]. Because death of syncytia by apoptosis is commonly observed in cocultures of infected and uninfected cells[57,58,59], cell-cell fusion is mainly considered to be a potential mechanism underlying $CD4^+$ T-cell depletion. Other reports argue that syncytia may function as viral reservoirs. Env-dependent cell fusion also has been implicated in the transfer of virus from infected cells to target cells through intercellular contacts, a form of infection that can be more efficient than infection by cell-free virus

**TABLE 1**
**Some Events Associated with Syncytia Formation in Pathogenic Viral Infections**

| Virus | Cell/Tissue | Observations | Ref. |
|---|---|---|---|
| Human herpes virus 6 | Hepatocytes | Infection induces giant cells that express viral antigens and induce the so-called syncytial giant-cell hepatitis. | [17] |
| Cytomegalovirus | Many tissues | Cell-cell fusion is a key mediator of viral spread.[a] | [18] |
| Respiratory syncytial virus (RSV) | Respiratory tract | Cell-cell fusion favors virus spread | [19] |
| | Epithelial cells | Low fusogenicity associates with persistence of RSV *in vitro.* | [20] |
| Measles virus (MV) | Epithelial cells of the respiratory tract | Virus spread from epithelial cells to underlying tissues occurs through cell fusion.[b] | [21] |
| | Epithelial and dendritic cells (DC) | Beta interferon (IFN-ß) production is amplified *in vitro* by virus-induced multinucleated giant cells derived from human epithelial cells or mature dendritic cells. | [22] |
| | DC and T lymphocytes | The interaction of MV-infected DC with T cells induces polykaryon formation and MV massive replication. | [23] |
| | Tracheobronchial lymph nodes and thymus | Live attenuated Edmonston strain induces large syncytia showing macrophage and DC surface markers in mice defective for the alpha/beta interferon receptor and expressing human CD46.[c] | [24] |
| Parainfluenza | Respiratory mucosal epithelial cells and diverse organs | Severe lung injury associated to highly reactive bronchial epithelial tissue containing multinucleated giant cells in patients with severe combined immunodeficiency (SCID).[d] | [25] |
| | Blood vessels | Antibodies directed against type 1 virus were shown in 40% of the cases vs. 20% of controls in giant-cell arteritis and polymyalgia rheumatica patients. | [26,27] |
| Murine leukemia virus (TR1.3 strain) | Endothelial capillary brain cells (ECBC) | Hemorrhagic disease with major brain damage associated to virus-induced syncytia.[e] | [28] |
| Epstein-Barr virus (EBV) | Lymphoid tissue DC | A clinicopathological study of 16 aggressive tumors containing EBV-positive Hodgkin and Reed-Sternberg-like giant cells. | [29] |
| Human papilloma virus 16 | Cervical epithelial cells | E5 viral protein induces binucleated cells by cell-cell fusion *in vitro*. Coexpression of E5 with E6/E7 viral oncogenes enhances proliferation of fused cells.[f] | [30] |
| Varicella zoster virus | Epidermis | Induction of cytopathic effect through syncytia induced by cell-cell fusion. | [31] |
| | Human dorsal root ganglion | Virus-induced cell-cell fusion and polykaryon formation between neurons and infected satellite cells during virus replication *in vivo*. Virus entry into neuronal cell bodies is necessary for its transfer to skin during herpes zoster. | [32] |

[a] This hypothesis is supported by the finding that, in spite of intense viral replication, free virus is rarely found in blood.

[b] Preventing virus-induced cell fusion abrogated the subepithelial spread of the virus *in vivo*.

[c] It is suggested that dissemination of MV in the initial stages of infection may occur by fusion of MV-infected monocytic cells with other cells.

[d] Syncytia were also detected in spleen, thymus, adrenal medulla, and urinary tract, among others tissues.

[e] Brain damage was associated exclusively with syncytia formation by ECBC cells.

[f] An interplay between E5, E6, and E7 proteins for the induction of carcinogenesis is suggested.

particles[60,61,62,63]. Other effects of syncytia formation, such as reduction in the rate of virus replication or promotion of recombination between virus strains, are also possible[64]. Table 2 contains a list of possible consequences of HIV-Env–dependent cell-cell fusion.

**TABLE 2**
**Possible Consequences of HIV-Induced Syncytia Formation**

| Consequence | Ref. |
|---|---|
| Cell death *in vitro* | [50,57,65,66] |
| T-cell depletion in blood | [44,67,68] |
| Spread by cell-to-cell transfer of virus at local fusion points | [61,63,69,70,71,72,73,74] |
| Reservoirs of replicating virus | [52,55,56,75,76,77] |
| Recombination of viruses from different strains | [64] |
| Enhancement of virus replication | [52,78] |
| Cell phenotype modification | N.f. |
| Survival of infected cells by fusion with healthy cells | N.f. |
| Reduction of number of cells in the microenvironment | N.f. |
| Reduction of virus replication | N.f. |
| Tissue injury by production of cytokines | N.f. |

*Note*: N.f., not found.

The extent of Env-mediated cell-cell fusion may be important in lymphoid organs, where the high cellular density favors close contact between cells. That infected cells may constitute a major source of infectious particles in HIV infection[23] emphasizes the necessity of a fuller understanding of the consequences of cell-cell fusion in the immune system. Such understanding would aid in approaches to the challenge of inhibiting the different forms of viral spreading and virus-induced cell damage.

## A METHOD FOR THE STUDY OF CELL-CELL FUSION BASED ON FLOW CYTOMETRY AND FLUORESCENCE RESONANCE ENERGY TRANSFER (FRET)

Cells transfected with the HIV *env* gene are a convenient system for the study of the mechanisms of HIV-induced membrane fusion and syncytia formation. In such cells, the gp160 precursor protein is synthesized, processed, and glycosylated, thus producing the mature gp120 and gp41 molecules that are then transported to the cell membrane and exposed to the extracellular milieu[79]. Stably transfected lymphocyte cell lines generated by Cao et al.[80] contain the *env* gene under the control of a tetracycline-inducible expression system, so that the synthesis of Env is repressed in the presence of tetracycline and is induced by the removal of the antibiotic from the medium ($Env^+$ cells). A second cell line (522F/Y cells) expresses a mutant, nonfusogenic Env protein[80] (Fig. 1). A few hours after $Env^+$ cells fused *in vitro* with target cells expressing the CD4 molecule and the proper coreceptor, a variety of fusion products can be detected (Fig. 2). Thus, because these cells provided an appropriate system for the study of HIV-Env syncytia formation, they were used in the experiments described in this article.

Usually, syncytia are counted by microscopy techniques or by the expression of reporter genes in the entire cell population[81,82,83]. We devised an approach for the quantitative study of HIV-Env–mediated cell-cell fusion, the aim of which was to integrate the study of the quantitative parameters (number and diversity of syncytia) of fusion with functional considerations and with cell-population dynamics during fusion[84,84a]. Our experimental approach was based on the staining of each of the fusion partners with one

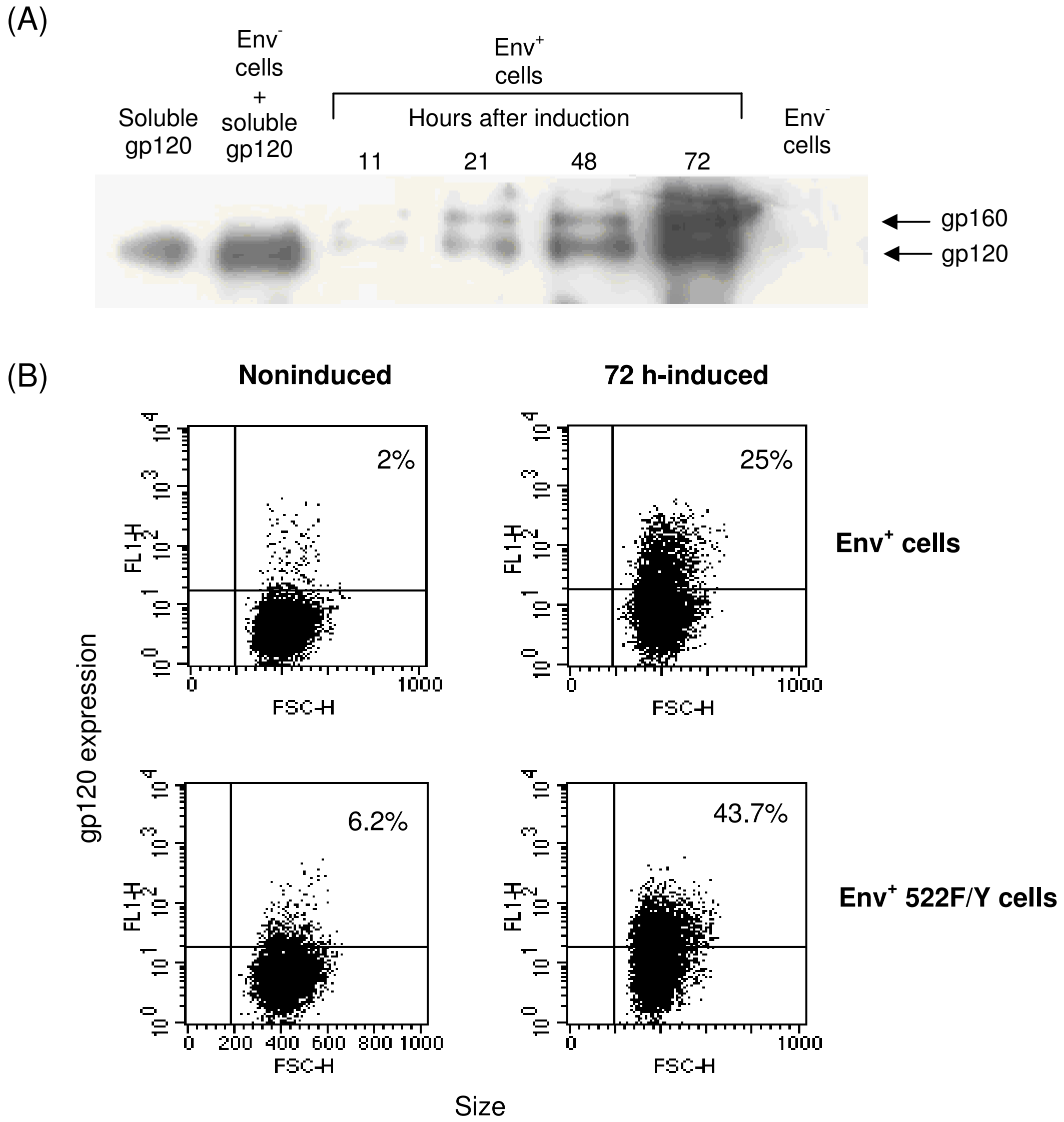

**FIGURE 1**. Tetracycline-inducible expression of HIV-Env proteins in Jurkat cells. Expression of the HIV-Env protein in transfected Jurkat cells was repressed when tetracycline was present in the culture medium (noninduced cells) and induced after its removal (induced cells). A time-dependent accumulation of envelope proteins in the induced cells was detected (A) by Western blot analysis of the immunoprecipitation products of cell extracts and (B) by flow cytometry on whole cells, using a rabbit anti-gp120 polyclonal antibody. In the Western blot, immunoprecipitates were separated by SDS-PAGE (7% gel) and transferred to a nitrocellulose membrane. After incubation with the anti-gp120 antibody and a second antibody coupled to horseradish peroxidase, the blot was then treated with a chemiluminescent peroxidase substrate. For the flow cytometry, a second antibody coupled to fluorescein isothiocyanate was used.

of the lipophilic fluorescent dyes, DiI (1,1'-dioctadecyl-3,3,3',3'-tetramethylindocarbocyanine perchlorate) and DiO (3,3'-dioctadecyloxacarbocyanine perchlorate), which produce red and green fluorescence when excited by a 488 laser beam, respectively[83]. $CD4^+$ cells were stained with DiI and $Env^+$ cells were stained with DiO. After coculture, the syncytia and those cells that did not fuse were quantified by flow cytometry as double- and single-colored particles, respectively. The relative morphological pattern of double- and single-fluorescent populations can be visualized in dot plots of the forward scatter signal (FSC-H) vs. the side scatter signal (SSC-H). These signals are a measure of the relative size and granularity of the cell populations, respectively. These plots evidenced the high heterogeneity of the fusion products (Fig. 3).

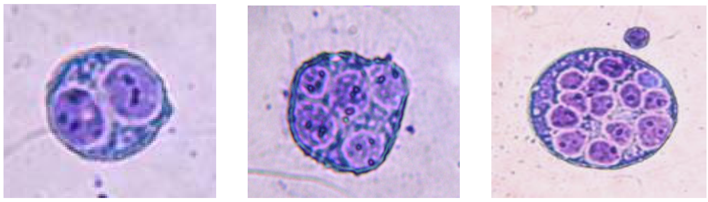

A B C

**FIGURE 2**. Heterogeneity of syncytia generated *in vitro* by the fusion of $Env^+$ and $CD4^+$ Jurkat cells in 5-h cocultures. (A) Binucleated cell; (B) five-nuclei cell; (C) multinucleated cell. Cells were stained with Giemsa.

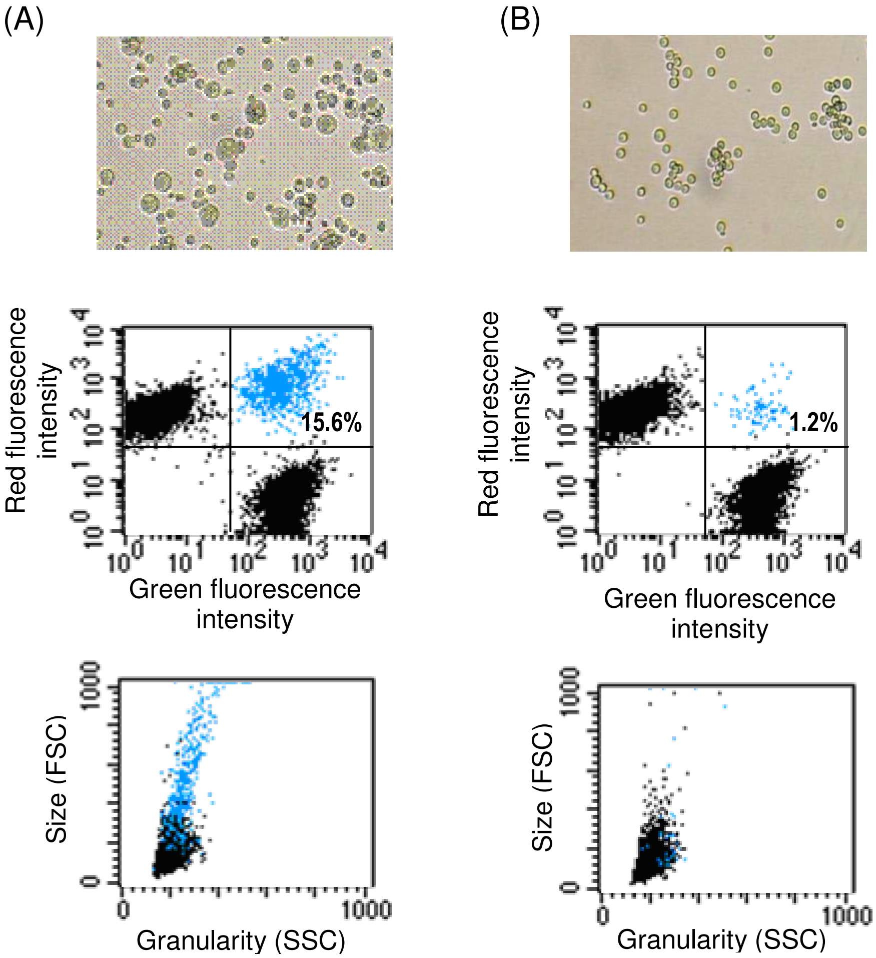

**FIGURE 3**. Microscopy view and flow cytometry analysis of syncytia formation in cocultures. Before coculture, $Env^+$ and $CD4^+$ cells were labeled with DiO and DiI, respectively. The cocultures of $CD4^+$ Jurkat cells either (A) with $Env^+$ fusogenic cells or (B) with $Env^+$ nonfusogenic cells (522F/Y) were viewed by light microscopy (10×) and analyzed by flow cytometry. The fluorescence and morphological patterns of cells after syncytia formation is shown in dot plots (FL1-H vs. FL2-H and FSC-H vs. SSC-H, respectively). Double-fluorescent particles are shown in blue and their percentage in the coculture is indicated.

Interaction between the two lipophilic dyes causes the DiI-red fluorescence to be enhanced due to FRET from DiO to DiI. This interaction is favored by the close proximity of the dyes in the fused cell membranes[85] and by the extensive overlap of the DiO-emission and DiI-absorption spectra (http://www.invitrogen.com/site/us/en/home/brands/Molecular-Probes.html). Here, double-fluorescent particles exhibited an enhancement of the DiI-red fluorescence, relative to that of nonfused DiI-stained $CD4^+$ cells (Fig. 3). Labeling of one of the fusion partners with increasing concentrations of DiO induced a proportional increase in the red fluorescence of the double-fluorescent population, indicating an additional excitation of the DiI molecule by DiO[87].

We also used cytoplasmic dyes 5-chloromethylfluorescein diacetate (CMFDA) and 5-(and-6)-(((4-chloromethyl)benzoyl)amino) tetramethylrhodamine (CMTMR) to label the fusion partners. However, because they did not show FRET, aggregated cells could not be distinguished from true fusions.

Enhancement of the red fluorescence in the double-fluorescent population was most easily observed when low concentrations of DiI and relatively high concentrations of DiO were used to label the cells. Two populations of double-fluorescent cells could be observed under these conditions: one was FRET-positive, showing approximately tenfold enhancement of the red fluorescence; the other, FRET-negative. An anti-CD4 monoclonal antibody, known to block CD4-gp120 interaction[86], inhibited the FRET-positive, but not the FRET-negative, cell population (Fig. 4A,B). Sorting and microscopic examination of these two populations showed the FRET-positive cells to be multinucleated, whereas the FRET-negative population was composed of single cells, indicating that they were cellular aggregates (Fig. 5)[87]. Thus, FRET between DiO and DiI allows the distinction and quantification of fused and aggregated cells, as determined by flow cytometry. FRET permits the detection of even small numbers of fused cells.

.

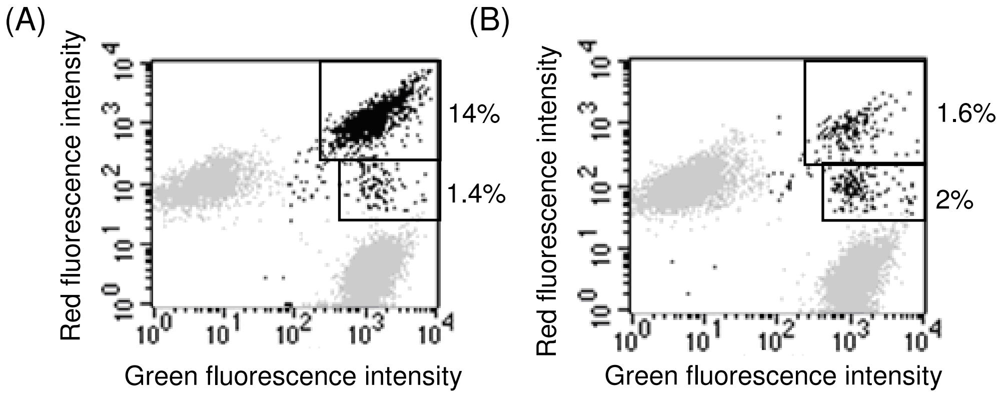

**FIGURE 4**. Fluorescence profiles of FRET-positive (fused cells, upper region) and FRET-negative (cellular aggregates, lower region) particles in cocultures of DiI-$CD4^+$ cells with (A) DiO-$Env^+$ cells and (B) DiO-$Env^+$ cells plus 30 µg/ml anti-CD4 monoclonal antibody. (Adapted from Huerta et al.[87]. Copyright 2006, with permission from Elsevier.)

Comparison of the number of syncytia counted by fluorescence microscopy and flow cytometry showed an excellent agreement between the two techniques, as shown in Table 3.

By using the FRET-based assay, a dose-dependent inhibition of fusion was obtained with monoclonal anti-CD4 and synthetic peptide T-20, which interferes with the membrane fusion step during virus entry[88]. The resulting values for the inhibition concentration ($IC_{50}$) agree with that obtained by other methods[89,90,91,92] (Fig. 6). All experiments described in this article were conducted in serum-free medium containing human albumin (AIM-V medium, Gibco) in order to avoid any interaction between, or combined effect of, components in fetal serum and the inhibitors tested.

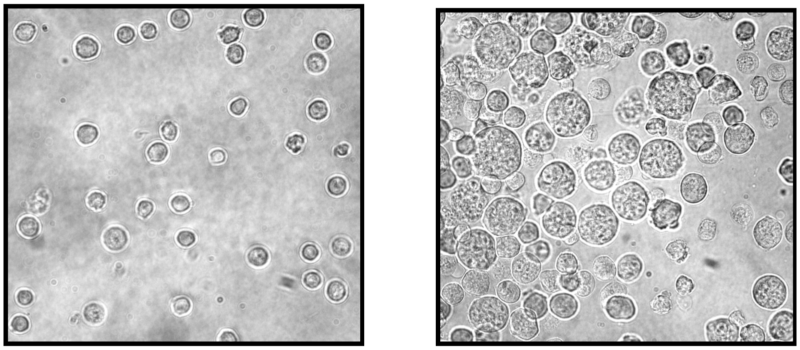

A B

**FIGURE 5.** Appearance of double-fluorescent cells after sorting of (A) FRET-negative and (B) FRET-positive particles. Magnification 400×. (Reprinted from Huerta et al.[87]. Copyright 2006, with permission from Elsevier.)

**TABLE 3**
**Percentage of Syncytia and Single Cells Obtained by Visual Counting and Flow Cytometry**

| | Microscopy Counting[a] | Flow Cytometry[b] |
|---|---|---|
| Single red (CD4+) | 45.1 | 41.3 |
| Single green (Env+) | 34 | 36.4 |
| Syncytia | 21 | 20.8 |

[a] Syncytia and single cells were identified as double- and single-fluorescent cells, respectively, in cocultures of DiI-CD4$^+$ and DiO-Env$^+$ cells. A total of 863 cells were analyzed in 58 microscope fields.

[b] FACS quantitation was carried out on 10,000 events.

Reprinted from Huerta et al.[87]. Copyright 2006, with permission from Elsevier.

## A QUANTITATIVE APPROACH TO THE STUDY OF SYNCYTIA DIVERSITY

Given the requirements for close contact and specific binding between cells, cell-cell fusion may roughly follow the principles of mass action. If so, the number of fusion reactions and the cellular composition of syncytia would depend on the duration of reaction, on the relative abundance of cell-fusion partners, and on the number of membrane receptors[93]. In turn, the extent of fusion (abundance of fused cells in a given compartment or microenvironment) and the cell composition of the syncytia (number and cell types incorporated into syncytia), in addition to the nature and intensity of stimuli from the microenvironment, must eventually determine the fate and biological significance of the syncytia. The study of cell-cell fusion by the flow cytometry method described in the previous section allows the quantitative analysis of several parameters of the cell-cell fusion reaction for a given experiment, i.e., the number of syncytia, average cell composition, and size distribution.

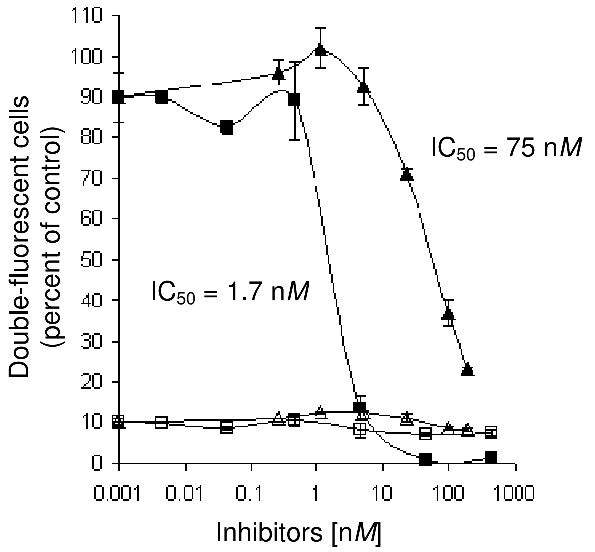

**FIGURE 6**. Effect of the fusion inhibitor peptide T-20 (squares) and anti-CD4 monoclonal antibody (triangles) on cell fusion (black symbols) and cellular aggregation (white symbols), as analyzed by the FRET-based flow cytometry method. Fused cells are sensitive to both inhibitors to different extents, while aggregated cells are not appreciably affected. (Reprinted from Huerta et al.[87]. Copyright 2006, with permission from Elsevier.)

The cellular composition of syncytia, i.e., the number of cells from each fusion partner that become fused, may prove to be an important biological parameter. For example, the ratio of healthy cells to virus-infected cells in a given syncytium may determine whether that syncytium will survive or die. In the FACS analysis shown above, it was possible both to identify and to count not only the syncytia, but also the cells that did not fuse (i.e., cells having only a single-colored fluorescence). Therefore, compared to the respective initial amounts of the fusion partners, changes in the number of nonfused cells could be used to calculate the number of cells incorporated into syncytia in short-term experiments, where cell proliferation or death are not significant. However, typical fluorometric analysis renders the proportion, but not the absolute number, of each cell type in the whole population. Given that changes in the proportion of cells remaining nonfused do not directly reflect the actual numbers of cells forming syncytia[84], the following simple expression was derived in order to calculate the $CD4^+/Env^+$ ratio of cells incorporated into syncytia, or fusion stoichiometry coefficient (here designated as *r*)[84], using the percentage data provided by most of FACS equipment available:

$$r_i = \frac{R_0 - (1 + R_0)\{cd4\}_i}{f_i}$$

In this expression, $R_0$, $\{cd4\}_i$, and $f_i$ correspond to the initial $CD4^+/Env^+$ ratio in the coculture, the percentage of nonfused $CD4^+$ cells, and the percentage of syncytia at time *i*, respectively. The fusion stoichiometry coefficient, *r*, has a value of 1 when the two fusion partners are equally incorporated into syncytia[84].

Fusion kinetics and the stoichiometry coefficient, *r*, can be calculated by determining the proportion of fused and nonfused cells in different culture wells as fusion develops, as shown for an 8-h coculture of Jurkat cells seeded at $R_0$ = 1 (Fig. 7A). In that study, maximal fusion ($F_{max}$) was reached in 4 h. The percent of nonfused cells diminished at similar rates, indicating that equivalent numbers of $CD4^+$ and

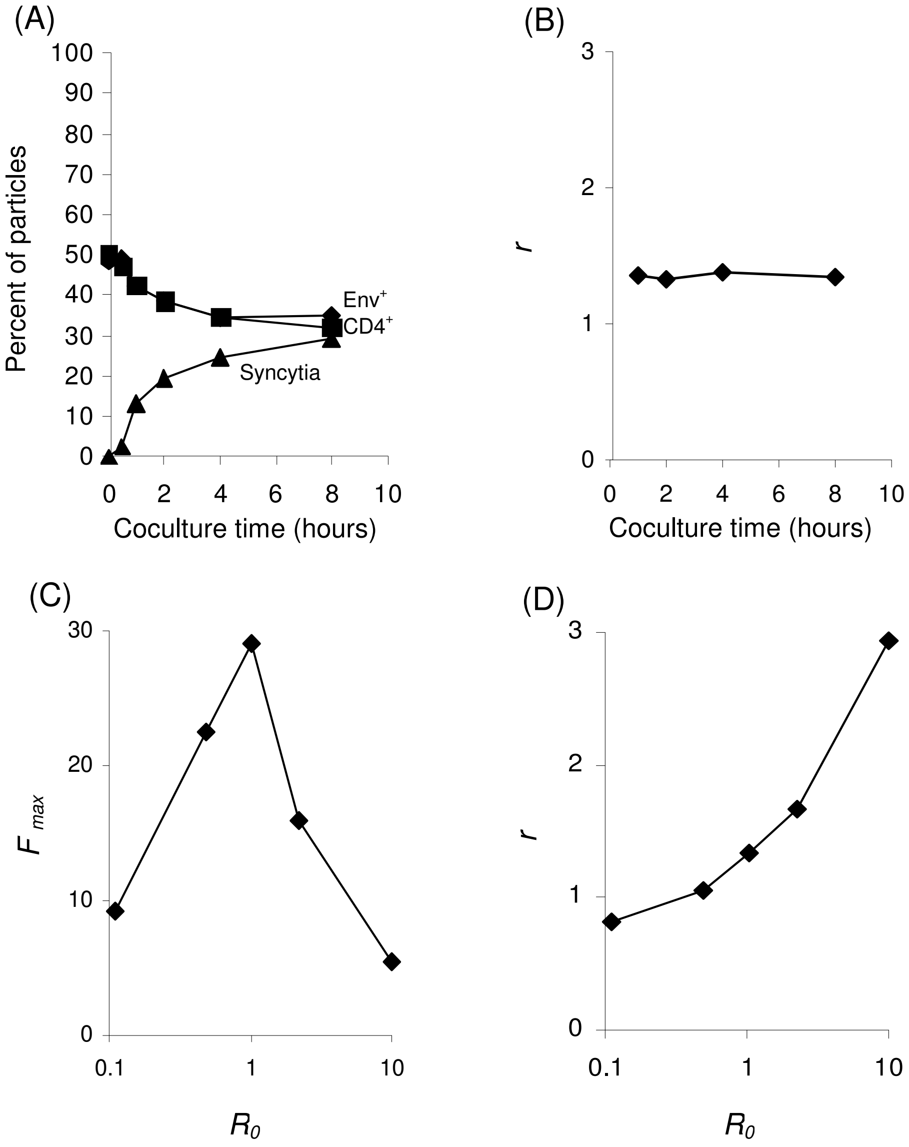

**FIGURE 7**. Changes in the percentages of syncytia and unfused cells (A) and in the ratio $r$ ($CD4^+/Env^+$) of incorporation of fusion partners into syncytia (B), at equal initial proportions of $CD4^+$ and $Env^+$ fusion partners ($R_0 = 1$). (C) Relationship of $F_{max}$ with $R_0$ and (D) relationship of $r$ with $R_0$. (Reprinted from Lopez-Balderas et al.[84]. Copyright 2007, with permission from Elsevier.)

$Env^+$ cells were incorporated into syncytia. In this example, $r$ showed a constant value of 1.4 throughout the duration of the culture (Fig. 7B).

At coculture ratios distinct from 1, $F_{max}$ decreased, probably due to the establishment of fewer effective contacts when one of the two fusion partners is in excess (Fig. 7C), while $r$ stabilized at different values, with a maximum of 3 at $R_0 = 10$ (Fig. 7D). Thus, the $CD4^+/Env^+$ ratio of cells incorporated into syncytia was 3:1 when $CD4^+$ cells were in tenfold excess, whereas this ratio was 0.7 (or 2:3 $CD4^+$ to $Env^+$ cells) when $Env^+$ were in tenfold excess. Thus, culture conditions can determine the generation of syncytia that differ in the abundance of one or the other cell type in their composition.

Distinct coculture conditions also generate different distributions of size and granularity of syncytia. Mean and standard deviations of the FSC-H and SSC-H parameters increase with coculture time and with $R_0$, so the largest syncytia were obtained with $R_0 = 10$, and the smallest with $R_0 = 0.1$ (Fig. 8). Overall, fused

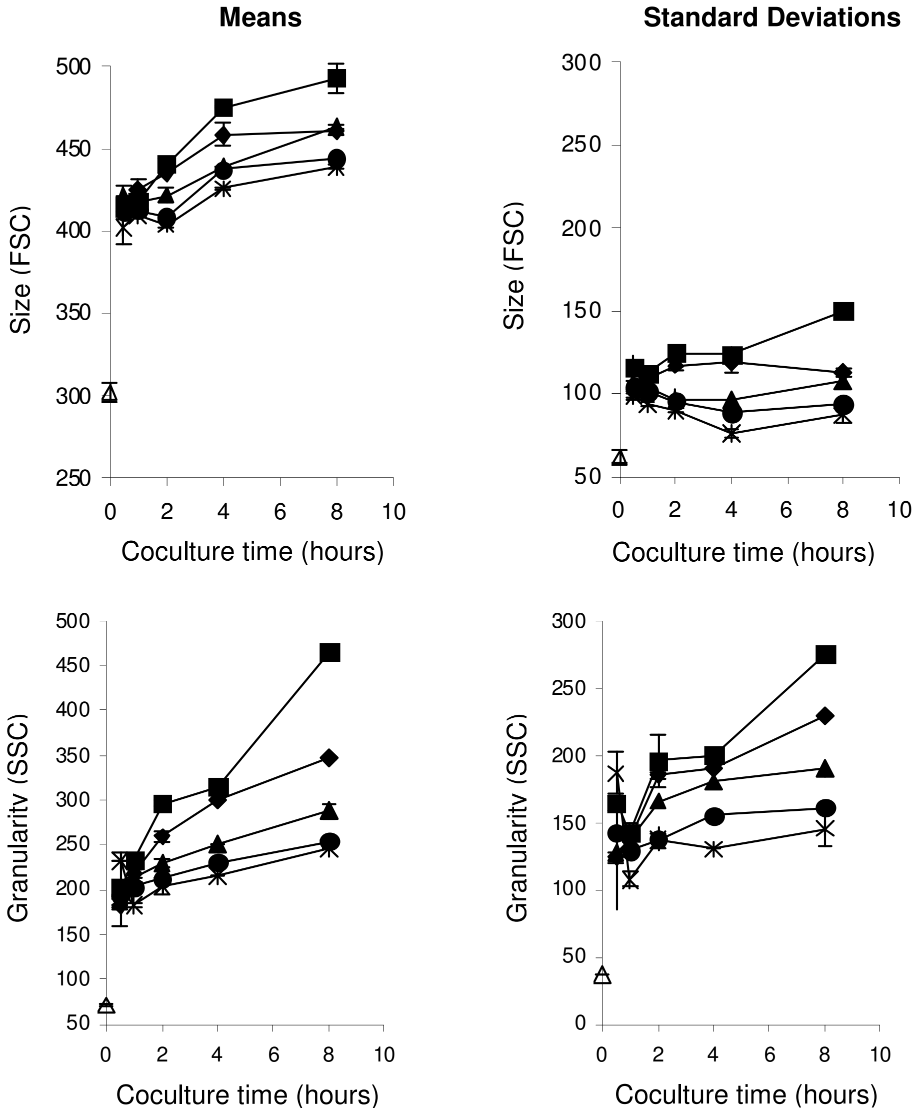

**FIGURE 8.** Changes in syncytia size (FSC) and granularity (SSC) with $R_0$ and time of coculture. ■, $R_0$ = 10; ◆, $R_0$ = 2.4; ▲, $R_0$ = 1; ●, $R_0$ = 0.5; ✶, $R_0$ = 0.1; △, unfused CD4$^+$cells. (Reprinted from Lopez-Balderas et al.[84]. Copyright 2007, with permission from Elsevier.)

cells show a major increase in granularity and a moderate increase in size, compared to nonfused cells. The modest increase in the intensity of the FSC-H signal of fused cells is related both to the observation that most of these cells were composed of two or three nuclei, as determined by microscopic counting (Fig. 9) and to the nonmonotonic nature of forward-scatter detector response. In contrast, the marked increase in the SSC-H signal is related to its high sensitivity to variations in the refractive index[94]. The finding that, under all the conditions tested, small syncytia comprised the greatest proportion (≈50%) points to the possibility that syncytia formed by only a few cells could be numerous during HIV infection and may go undetected in analysis by microscopy.

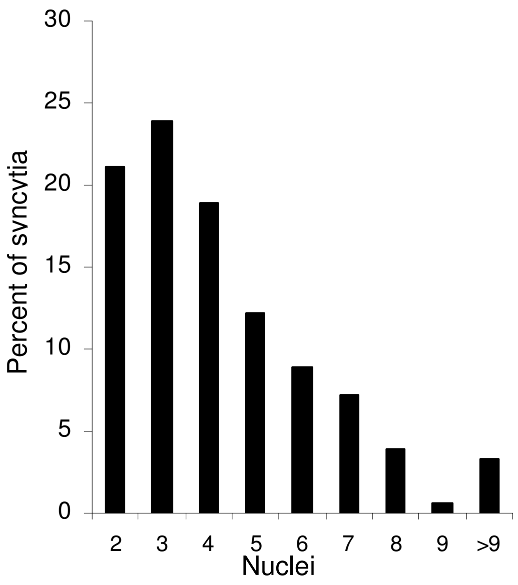

**FIGURE 9.** (A) Number of nuclei in syncytia formed in cocultures of DiI-CD4$^+$ and DiO-Env$^+$ cells. Nuclei were stained with the Hoechst 33342 dye and counted in 180 triple-fluorescent cells. (Reprinted from Huerta et al.[87]. Copyright 2006, with permission from Elsevier.)

Thus, as shown by the experimental results described above, the extent of fusion, the stoichiometry coefficient, and size of syncytia vary with time of reaction and initial proportion of fusion partners. The extent of fusion and diversity of syncytia under different experimental conditions may reflect heterogeneous features of the fusion-partner cell populations, such as different levels of expression of fusion-active proteins, or asynchronous cell cycles among the cell-line members. On the other hand, the increase in syncytia size without a similar increase of the proportion of fused cells suggests the occurrence of new fusion events that do not increase the number of double-fluorescent particles, such as the fusion of cells into already formed syncytia or fusion among syncytia.

## ASSOCIATION OF EFFECT OF SERA ON FUSION WITH MARKERS OF HIV DISEASE

Cell-cell fusion and cell-to-cell spreading of HIV may be affected by antibodies produced by HIV-infected patients. Although those antibodies that neutralize the cell-free virus would also block the Env-dependent cell-cell fusion, several findings indicate that virus-cell and cell-cell fusion may have different requirements[69,95]. Contact between extensive areas of the membranes of cells would involve interactions among large numbers of receptors and adhesion molecules[96]. Thus, blocking of Env-dependent cell-cell fusion may require higher concentrations of antibodies than those necessary to neutralize virus particles[97,98,99,100,101,102]. This idea is based on the finding that, in experiments using monoclonal antibodies against epitopes on the proteins gp120 and gp41, the concentration of monoclonal antibodies needed to inhibit fusion between Env$^+$ and CD4$^+$ Jurkat cells (Fig. 10) was considerably higher than those reported for neutralization of cell-free virus[98,99,100,101].

We explored the effect of sera from HIV-infected individuals on the fusion of Env$^+$ and CD4$^+$ Jurkat cells[103]. Most (69%) of the sera from a sampling of 49 HIV-positive individuals inhibited fusion to some extent; 24.5% had no effect and 6.1% enhanced fusion. Inhibition of fusion correlated with the asymptomatic stage of human HIV infection, whereas sera that had no effect or enhanced fusion were

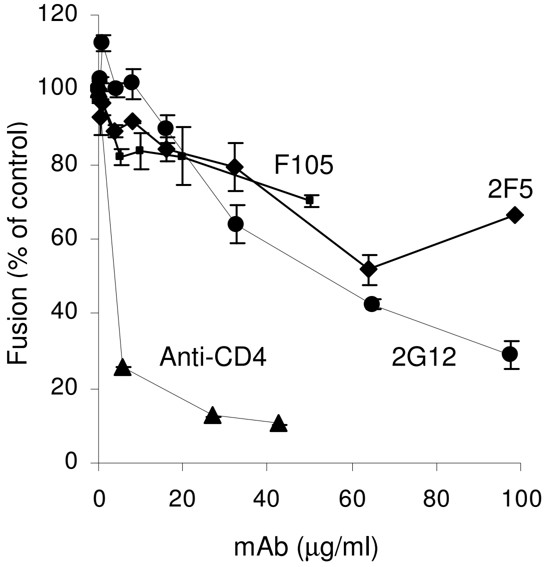

**FIGURE 10**. Fusion inhibition obtained with anti-CD4, anti-gp41 (2F5), and two anti-gp120 (F105 and 2G12) monoclonal antibodies. Data are expressed as percent of fusion obtained in the absence of antibodies. (Reprinted from Huerta et al.[103]. Copyright 2005, with permission from the Society for General Microbiology.)

associated with AIDS (Fig. 11). Coculture of labeled cells was performed in the absence of fetal bovine sera in order to avoid the influence of stimulating factors in the fetal serum on the patient's sera effect on fusion. The inhibition and enhancement of fusion by the patients' sera were shown to be related to IgG and IgM antibodies, respectively[103]. This study indicates that fusion-inhibiting antibodies predominate in the early stages of the disease, while loss of inhibition and the presence of fusion-enhancing antibodies appear during the late stages. This underscores the point that the effect of antibodies on syncytia formation should be considered in order to understand fully the role of the immune response in the control of the different forms of viral spreading and cell damage.

## CONCLUDING REMARKS

Cell-cell fusion is a highly heterogeneous process; it results in diverse outcomes having potentially significant consequences for the success or failure of the HIV infection. Studies of the biological features of syncytia must deal with such diversity in order to form an objective picture of their biological significance *in vivo*. Given the heterogeneity of fusion products, it is probable that not all the possible outcomes of cell-cell fusion induced by HIV infection have been investigated. A possibility to be considered is that, although multiple cell-cell fusion events lead to formation of multinucleated giant cells, there may be many fused cells *in vivo* too small to be easily detected by current methods.

Flow cytometry provides a convenient tool for the quantitative study of cell-cell fusion. The use of fluorescent dyes and of flow cytometry allows the high-speed analysis of the number, size, and average cellular composition of syncytia. This technique can be used not only to distinguish between aggregated and *bona fide* fused cells, but also to test for potential inhibitors to HIV entry. DiI and DiO can be combined with third-party fluorescent dyes coupled to antibodies for the determination of phenotypic markers of syncytia and other biological features (i.e., mechanism of death, cell cycle, cytokine production, virus production capability). The same features also can be determined in the surrounding cells that do not fuse.

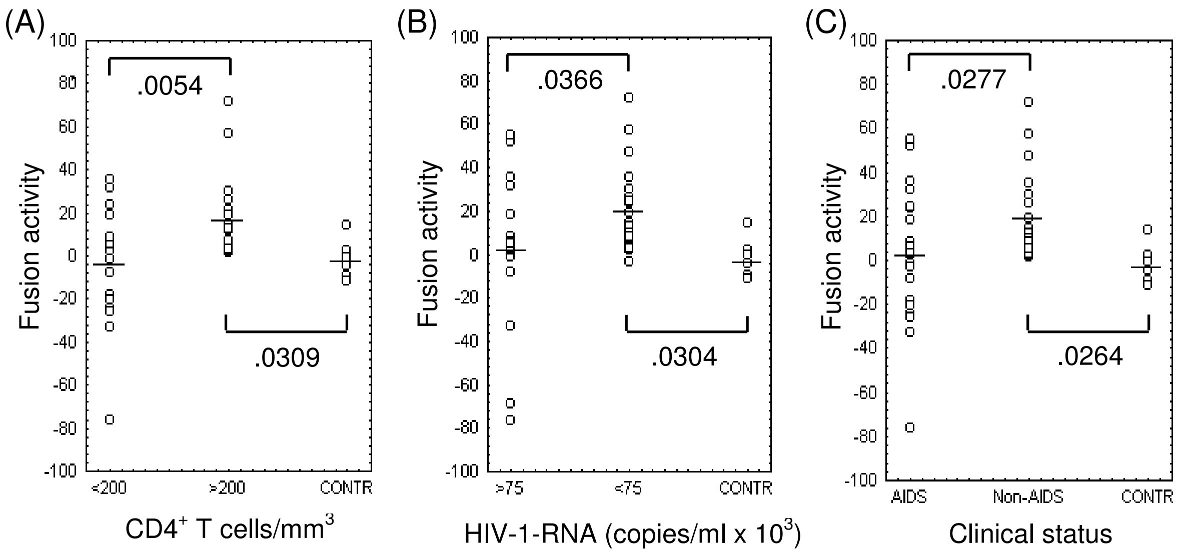

**FIGURE 11**. Relationship of sera activity on fusion with CD4+ lymphocyte count (A), viral load (B), and clinical status (C). Values of fusion activity greater and lower than zero indicate inhibition or enhancement of fusion, respectively. Probability values (*p*) for significant differences are shown. Dilution of sera was 1:50. CONTR: HIV-negative controls. (Reprinted from Huerta et al.[103]. Copyright 2005, with permission from the Society for General Microbiology.)

The combination of flow cytometry and FRET provides a method with sensitivity similar in degree to that of other current methodologies. It also has the advantages of technical simplicity, minimal cell-invasive procedures, and the possibility of using additional, distinct fluorochromes to explore simultaneously other biological markers of the cells involved in fusion.

## ACKNOWLEDGMENTS

Env+ cells (HXBc2 and 522F/Y Jurkat cells) were obtained from Dr. Joseph Sodroski, and CD4+ cells (Jurkat Clone E6-1) from ATCC (Dr. Arthur Weiss), through the AIDS Research and Reference Reagent Program, Division of AIDS, NIAID, NIH. This work was supported by Consejo Nacional de Ciencia y Tecnología of México (CONACYT; Grant 61425) and by the Dirección General de Asuntos del Personal Académico, Universidad Nacional Autónoma de México (PAPIIT; Grant IN229307). We thank Elsevier and the Society for General Microbiology for allowing us to reproduce previously published figures. The authors thank Veronica Yakoleff for editing, and Dr. Nuno C. Santos, editor of this issue of *TheScientificWorldJOURNAL*, for handling the manuscript.

## References

1. Horsley, V., Jansen, K.M., Mills, S.T., and Pavlath, G.K. (2003) IL-4 acts as a myoblast recruitment factor during mammalian muscle growth. *Cell* **113**, 483–494.
2. Huppertz, B., Bartz, C., and Kokozidou, M. (2006) Trophoblast fusion: fusogenic proteins, syncytins and ADAMs, and other prerequisites for syncytial fusion. *Micron* **37**, 509–517.
3. Shemer, G. and Podbilewicz, B. (2003) The story of cell fusion: big lessons from little worms. *Bioessays* **25**, 672–682.
4. Alvarez-Dolado, M., Pardal, R., Garcia-Verdugo, J.M., Fike, J.R., Lee, H.O., Pfeffer, K., Lois, C., Morrison, S.J., and Alvarez-Buylla, A. (2003) Fusion of bone-marrow-derived cells with Purkinje neurons, cardiomyocytes and hepatocytes. *Nature* **425**, 968–973.

5. Camargo, F.D., Green, R., Capetanaki, Y., Jackson, K.A., and Goodell, M.A. (2003) Single hematopoietic stem cells generate skeletal muscle through myeloid intermediates. *Nat. Med.* **9**, 1520–1527.
6. Rodic, N., Rutenberg, M.S., and Terada, N. (2004) Cell fusion and reprogramming: resolving our transdifferences. *Trends Mol. Med.* **10**, 93–96.
7. Matsuura, K., Wada, H., Nagai, T., Iijima, Y., Minamino, T., Sano, M., Akazawa, H., Molkentin, J.D., Kasanuki, H., and Komuro, I. (2004) Cardiomyocytes fuse with surrounding noncardiomyocytes and reenter the cell cycle. *J. Cell Biol.* **167**, 351–363.
8. Duelli, D. and Lazebnik, Y. (2003) Cell fusion: a hidden enemy? *Cancer Cell* **3**, 445–448.
9. Duelli, D. and Lazebnik, Y. (2007) Cell-to-cell fusion as a link between viruses and cancer. *Nat. Rev. Cancer* **7**, 968–976.
10. Ogle, B.M., Cascalho, M., and Platt, J.L. (2005) Biological implications of cell fusion. *Nat. Rev. Mol. Cell Biol.* **6**, 567–575.
11. Vignery, A. (2005) Macrophage fusion: the making of osteoclasts and giant cells. *J. Exp. Med.* **202**, 337–340.
12. Anderson, J.M., Rodriguez, A., and Chang, D.T. (2008) Foreign body reaction to biomaterials. *Semin. Immunol.* **20**, 86–100.
13. Blau, H.M. and Blakely, B.T. (1999) Plasticity of cell fate: insights from heterokaryons. *Semin. Cell Dev. Biol.* **10**, 267–272.
14. Terada, N., Hamazaki, T., Oka, M., Hoki, M., Mastalerz, D.M., Nakano, Y., Meyer, E.M., Morel, L., Petersen, B.E., and Scott, E.W. (2002) Bone marrow cells adopt the phenotype of other cells by spontaneous cell fusion. *Nature* **416**, 542–545.
15. Ying, Q.L., Nichols, J., Evans, E.P., and Smith, A.G. (2002) Changing potency by spontaneous fusion. *Nature* **416**, 545–548.
16. Sattentau, Q. (2008) Avoiding the void: cell-to-cell spread of human viruses. *Nat. Rev. Microbiol.* **6**, 815–826.
17. Potenza, L., Luppi, M., Barozzi, P., Rossi, G., Cocchi, S., Codeluppi, M., Pecorari, M., Masetti, M., Di Benedetto, F., Gennari, W., Portolani, M., Gerunda, G.E., Lazzarotto, T., Landini, M.P., Schulz, T.F., Torelli, G., and Guaraldi, G. (2008) HHV-6A in syncytial giant-cell hepatitis. *N. Engl. J. Med.* **359**, 593–602.
18. Sweet, C. (1999) The pathogenicity of cytomegalovirus. *FEMS Microbiol. Rev.* **23**, 457–482.
19. Collins, P.L. and Crowe, J.E. (2007) Respiratory syncytial virus and metaneumovirus. In *Field's Virology*. 5th ed. Knipe, D.M. and Howley, P.M., Eds. Lippincott Williams & Wilkins, Philadelphia. pp. 1601–1646.
20. Sarmiento, R.E., Arias, C.F., Mendez, E., and Gomez, B. (2009) Characterization of a persistent respiratory syncytial virus showing a low-fusogenic activity associated to an impaired F protein. *Virus Res.* **139**, 39–47.
21. Moll, M., Pfeuffer, J., Klenk, H.D., Niewiesk, S., and Maisner, A. (2004) Polarized glycoprotein targeting affects the spread of measles virus in vitro and in vivo. *J. Gen. Virol.* **85**, 1019–1027.
22. Herschke, F., Plumet, S., Duhen, T., Azocar, O., Druelle, J., Laine, D., Wild, T.F., Rabourdin-Combe, C., Gerlier, D., and Valentin, H. (2007) Cell-cell fusion induced by measles virus amplifies the type I interferon response. *J. Virol.* **81**, 12859–12871.
23. Fugier-Vivier, I., Servet-Delprat, C., Rivailler, P., Rissoan, M.C., Liu, Y.J., and Rabourdin-Combe, C. (1997) Measles virus suppresses cell-mediated immunity by interfering with the survival and functions of dendritic and T cells. *J. Exp. Med.* **186**, 813–823.
24. Mrkic, B., Odermatt, B., Klein, M.A., Billeter, M.A., Pavlovic, J., and Cattaneo, R. (2000) Lymphatic dissemination and comparative pathology of recombinant measles viruses in genetically modified mice. *J. Virol.* **74**, 1364–1372.
25. Madden, J.F., Burchette, J.L., Jr., and Hale, L.P. (2004) Pathology of parainfluenza virus infection in patients with congenital immunodeficiency syndromes. *Hum. Pathol.* **35**, 594–603.
26. Duhaut, P., Bosshard, S., and Dumontet, C. (2000) Giant cell arteritis and polymyalgia rheumatica: role of viral infections. *Clin. Exp. Rheumatol.* **18**, S22–23.
27. Duhaut, P., Bosshard, S., and Ducroix, J.P. (2004) Is giant cell arteritis an infectious disease? Biological and epidemiological evidence. *Presse Med.* **33**, 1403–1408.
28. Landers, M.C., Dugger, N., Quadros, M., Hoffman, P.M., and Gaulton, G.N. (2004) Neuropathogenic murine leukemia virus TR1.3 induces selective syncytia formation of brain capillary endothelium. *Virology* **321**, 57–64.
29. Shimazaki, K., Ohshima, K., Haraoka, S., Suzumiya, J., Nakamura, N., and Kikuchi, M. (2002) Accessory cell tumour: a clinicopathological study of 16 aggressive tumours containing EBV-positive Hodgkin and Reed-Sternberg-like giant cells. *Histopathology* **40**, 12–21.
30. Hu, L., Plafker, K., Vorozhko, V., Zuna, R.E., Hanigan, M.H., Gorbsky, G.J., Plafker, S.M., Angeletti, P.C., and Ceresa, B.P. (2009) Human papillomavirus 16 E5 induces bi-nucleated cell formation by cell-cell fusion. *Virology* **384**, 125–134.
31. Cole, N.L. and Grose, C. (2003) Membrane fusion mediated by herpesvirus glycoproteins: the paradigm of varicella-zoster virus. *Rev. Med. Virol.* **13**, 207–222.
32. Reichelt, M., Zerboni, L., and Arvin, A.M. (2008) Mechanisms of varicella-zoster virus neuropathogenesis in human dorsal root ganglia. *J. Virol.* **82**, 3971–3983.
33. Cantin, R., Methot, S., and Tremblay, M.J. (2005) Plunder and stowaways: incorporation of cellular proteins by enveloped viruses. *J. Virol.* **79**, 6577–6587.
34. Pantaleo, G., Butini, L., Graziosi, C., Poli, G., Schnittman, S.M., Greenhouse, J.J., Gallin, J.I., and Fauci, A.S. (1991)

Human immunodeficiency virus (HIV) infection in CD4+ T lymphocytes genetically deficient in LFA-1: LFA-1 is required for HIV-mediated cell fusion but not for viral transmission. *J. Exp. Med*. **173**, 511–514.

35. Bastiani Lallos, L., Cecilia, D., Fenyo, E.M., Laal, S., and Zolla-Pazner, S. (2000) HIV phenotype correlates with the relative amounts of lymphocyte function-related molecule 1 (LFA-1) and major histocompatibility complex (MHC) class II in the virion envelope. *AIDS* **14**, 1523–1531.
36. Fortin, J.F., Cantin, R., Bergeron, M.G., and Tremblay, M.J. (2000) Interaction between virion-bound host intercellular adhesion molecule-1 and the high-affinity state of lymphocyte function-associated antigen-1 on target cells renders R5 and X4 isolates of human immunodeficiency virus type 1 more refractory to neutralization. *Virology* **268**, 493–503.
37. Hernandez, L.D., Hoffman, L.R., Wolfsberg, T.G., and White, J.M. (1996) Virus-cell and cell-cell fusion. *Annu. Rev. Cell Dev. Biol*. **12**, 627–661.
38. Lentz, B.R., Malinin, V., Haque, M.E., and Evans, K. (2000) Protein machines and lipid assemblies: current views of cell membrane fusion. *Curr. Opin. Struct. Biol*. **10,** 607–615.
39. Chernomordik, L.V. and Kozlov, M.M. (2008) Mechanics of membrane fusion. *Nat. Struct. Mol. Biol.* **15**, 675–683.
40. Melikyan, G.B. (2008) Common principles and intermediates of viral protein-mediated fusion: the HIV-1 paradigm. *Retrovirology* **5**, 111.
41. Chen, E.H. and Olson, E.N. (2005) Unveiling the mechanisms of cell-cell fusion. *Science* **308**, 369–373.
42. Gallo, S.A., Finnegan, C.M., Viard, M., Raviv, Y., Dimitrov, A., Rawat, S.S., Puri, A., Durell, S., and Blumenthal, R. (2003) The HIV Env-mediated fusion reaction. *Biochim. Biophys. Acta* **1614**, 36–50.
43. Lifson, J.D., Feinberg, M.B., Reyes, G.R., Rabin, L., Banapour, B., Chakrabarti, S., Moss, B., Wong-Staal, F., Steimer, K.S., and Engleman, E.G. (1986) Induction of CD4-dependent cell fusion by the HTLV-III/LAV envelope glycoprotein. *Nature* **323**, 725–728.
44. Lifson, J.D., Reyes, G.R., McGrath, M.S., Stein, B.S., and Engleman, E.G. (1986) AIDS retrovirus induced cytopathology: giant cell formation and involvement of CD4 antigen. *Science* **232**, 1123–1127.
45. Sodroski, J., Goh, W.C., Rosen, C., Campbell, K., and Haseltine, W.A. (1986) Role of the HTLV-III/LAV envelope in syncytium formation and cytopathicity. *Nature* **322**, 470–474.
46. Connor, R.I., Mohri, H., Cao, Y., and Ho, D.D. (1993) Increased viral burden and cytopathicity correlate temporally with CD4+ T-lymphocyte decline and clinical progression in human immunodeficiency virus type 1-infected individuals. *J. Virol*. **67**, 1772–1777.
47. Blaak, H., van't Wout, A.B., Brouwer, M., Hooibrink, B., Hovenkamp, E., and Schuitemaker, H. (2000) In vivo HIV-1 infection of CD45RA(+)CD4(+) T cells is established primarily by syncytium-inducing variants and correlates with the rate of CD4(+) T cell decline. *Proc. Natl. Acad. Sci. U. S. A.* **97**, 1269–1274.
48. Miedema, F., Meyaard, L., Koot, M., Klein, M.R., Roos, M.T., Groenink, M., Fouchier, R.A., Van't Wout, A.B., Tersmette, M., Schellekens, P.T., and Schuitemaker, H. (1994) Changing virus-host interactions in the course of HIV-1 infection. *Immunol. Rev.* **140,** 35–72.
49. Furrer, H., Wendland, T., Minder, C., Christen, A., von Overbeck, J., Grunow, R., Pichler, W., and Malinverni, R.P. (1998) Association of syncytium-inducing phenotype of HIV-1 with CD4 cell count, viral load and sociodemographic characteristics. *AIDS* **12**, 1341–1346.
50. Amendola, A., Gougeon, M.L., Poccia, F., Bondurand, A., Fesus, L., and Piacentini, M. (1996) Induction of "tissue" transglutaminase in HIV pathogenesis: evidence for high rate of apoptosis of CD4+ T lymphocytes and accessory cells in lymphoid tissues. *Proc. Natl. Acad. Sci. U. S. A.* **93**, 11057–11062.
51. Budka, H. (1986) Multinucleated giant cells in brain: a hallmark of the acquired immune deficiency syndrome (AIDS). *Acta Neuropathol*. **69**, 253–258.
52. Frankel, S.S., Wenig, B.M., Burke, A.P., Mannan, P., Thompson, L.D., Abbondanzo, S.L., Nelson, A.M., Pope, M., and Steinman, R.M. (1996) Replication of HIV-1 in dendritic cell-derived syncytia at the mucosal surface of the adenoid. *Science* **272**, 115–117.
53. Koenig, S., Gendelman, H.E., Orenstein, J.M., Dal Canto, M.C., Pezeshkpour, G.H., Yungbluth, M., Janotta, F., Aksamit, A., Martin, M.A., and Fauci, A.S. (1986) Detection of AIDS virus in macrophages in brain tissue from AIDS patients with encephalopathy. *Science* **233**, 1089–1093.
54. Orenstein, J.M. (2000) In vivo cytolysis and fusion of human immunodeficiency virus type 1-infected lymphocytes in lymphoid tissue. *J. Infect. Dis*. **182**, 338–342.
55. Rinfret, A., Latendresse, H., Lefebvre, R., St-Louis, G., Jolicoeur, P., and Lamarre, L. (1991) Human immunodeficiency virus-infected multinucleated histiocytes in oropharyngeal lymphoid tissues from two asymptomatic patients. *Am. J. Pathol*. **138**, 421–426.
56. Bhoopat, L., Rithaporn, T.S., Khunamornpong, S., Bhoopat, T., Taylor, C.R., and Thorner, P.S. (2006) Cell reservoirs in lymph nodes infected with HIV-1 subtype E differ from subtype B: identification by combined in situ polymerase chain reaction and immunohistochemistry. *Mod. Pathol*. **19**, 255–263.
57. Castedo, M., Perfettini, J.L., Andreau, K., Roumier, T., Piacentini, M., and Kroemer, G. (2003) Mitochondrial apoptosis induced by the HIV-1 envelope. *Ann. N. Y. Acad. Sci.* **1010**, 19–28.
58. Ferri, K.F., Jacotot, E., Blanco, J., Este, J.A., Zamzami, N., Susin, S.A., Xie, Z., Brothers, G., Reed, J.C., Penninger, J.M., and Kroemer, G. (2000) Apoptosis control in syncytia induced by the HIV type 1-envelope glycoprotein complex: role of mitochondria and caspases. *J. Exp. Med*. **192**, 1081–1092.

59. Perfettini, J.L., Castedo, M., Roumier, T., Andreau, K., Nardacci, R., Piacentini, M., and Kroemer, G. (2005) Mechanisms of apoptosis induction by the HIV-1 envelope. *Cell Death Differ.* **12(Suppl 1),** 916–923.
60. Chen, P., Hubner, W., Spinelli, M.A., and Chen, B.K. (2007) Predominant mode of human immunodeficiency virus transfer between T cells is mediated by sustained Env-dependent neutralization-resistant virological synapses. *J. Virol.* **81**, 12582–12595.
61. Dimitrov, D.S., Willey, R.L., Sato, H., Chang, L.J., Blumenthal, R., and Martin, M.A. (1993) Quantitation of human immunodeficiency virus type 1 infection kinetics. *J. Virol.* **67**, 2182–2190.
62. Lagaye, S., Derrien, M., Menu, E., Coito, C., Tresoldi, E., Mauclere, P., Scarlatti, G., Chaouat, G., Barre-Sinoussi, F., and Bomsel, M. (2001) Cell-to-cell contact results in a selective translocation of maternal human immunodeficiency virus type 1 quasispecies across a trophoblastic barrier by both transcytosis and infection. *J. Virol.* **75**, 4780–4791.
63. Sato, H., Orenstein, J., Dimitrov, D., and Martin, M. (1992) Cell-to-cell spread of HIV-1 occurs within minutes and may not involve the participation of virus particles. *Virology* **186**, 712–724.
64. Steain, M.C., Wang, B., and Saksena, N.K. (2008) The possible contribution of HIV-1-induced syncytia to the generation of intersubtype recombinants in vitro. *AIDS* **22**, 1009–1017.
65. Ferri, K.F., Jacotot, E., Geuskens, M., and Kroemer, G. (2000) Apoptosis and karyogamy in syncytia induced by the HIV-1-envelope glycoprotein complex. *Cell Death Differ.* **7**, 1137–1139.
66. Nardacci, R., Antinori, A., Larocca, L.M., Arena, V., Amendola, A., Perfettini, J.L., Kroemer, G., and Piacentini, M. (2005) Characterization of cell death pathways in human immunodeficiency virus-associated encephalitis. *Am. J. Pathol.* **167**, 695–704.
67. Karlsson, G.B., Halloran, M., Li, J., Park, I.W., Gomila, R., Reimann, K.A., Axthelm, M.K., Iliff, S.A., Letvin, N.L., and Sodroski, J. (1997) Characterization of molecularly cloned simian-human immunodeficiency viruses causing rapid CD4+ lymphocyte depletion in rhesus monkeys. *J. Virol.* **71**, 4218–4225.
68. LaBonte, J.A., Patel, T., Hofmann, W., and Sodroski, J. (2000) Importance of membrane fusion mediated by human immunodeficiency virus envelope glycoproteins for lysis of primary CD4-positive T cells. *J. Virol.* **74**, 10690–10698.
69. Gupta, P., Balachandran, R., Ho, M., Enrico, A., and Rinaldo, C. (1989) Cell-to-cell transmission of human immunodeficiency virus type 1 in the presence of azidothymidine and neutralizing antibody. *J. Virol.* **63**, 2361–2365.
70. Phillips, D.M. and Bourinbaiar, A.S. (1992) Mechanism of HIV spread from lymphocytes to epithelia. *Virology* **186**, 261–273.
71. Sun, J., Barbeau, B., and Tremblay, M.J. (2006) HIV-1-mediated syncytium formation promotes cell-to-cell transfer of Tax protein and HTLV-I gene expression. *Virus Res.* **118**, 120–129.
72. Carr, J.M., Hocking, H., Li, P., and Burrell, C.J. (1999) Rapid and efficient cell-to-cell transmission of human immunodeficiency virus infection from monocyte-derived macrophages to peripheral blood lymphocytes. *Virology* **265**, 319–329.
73. Sherer, N.M., Lehmann, M.J., Jimenez-Soto, L.F., Horensavitz, C., Pypaert, M., and Mothes, W. (2007) Retroviruses can establish filopodial bridges for efficient cell-to-cell transmission. *Nat. Cell Biol.* **9**, 310–315.
74. Ruggiero, E., Bona, R., Muratori, C., and Federico, M. (2008) Virological consequences of early events following cell-cell contact between human immunodeficiency virus type 1-infected and uninfected CD4+ cells. *J. Virol.* **82**, 7773–7789.
75. Ioannidis, J.P., Reichlin, S., and Skolnik, P.R. (1995) Long-term productive human immunodeficiency virus-1 infection in human infant microglia. *Am. J. Pathol.* **147**, 1200–1206.
76. Fais, S., Borghi, P., Gherardi, G., Logozzi, M., Belardelli, F., and Gessani, S. (1996) Human immunodeficiency virus type 1 induces cellular polarization, intercellular adhesion molecule-1 redistribution, and multinucleated giant cell generation in human primary monocytes but not in monocyte-derived macrophages. *Lab. Invest.* **75**, 783–790.
77. Jimenez, J.L., Sanchez-Ramon, S., Palomino, S.S., Alcami, J., and Munoz-Fernandez, H.A. (2001) Where does free infective HIV-1 rebound come from? *AIDS* **15**, 657.
78. Li, H., Haviv, Y.S., Derdeyn, C.A., Lam, J., Coolidge, C., Hunter, E., Curiel, D.T., and Blackwell, J.L. (2001) Human immunodeficiency virus type 1-mediated syncytium formation is compatible with adenovirus replication and facilitates efficient dispersion of viral gene products and de novo-synthesized virus particles. *Hum. Gene Ther.* **12**, 2155–2165.
79. Kozarsky, K., Penman, M., Basiripour, L., Haseltine, W., Sodroski, J., and Krieger, M. (1989) Glycosylation and processing of the human immunodeficiency virus type 1 envelope protein. *J. Acquir. Immune Defic. Syndr.* **2**, 163–169.
80. Cao, J., Park, I.W., Cooper, A., and Sodroski, J. (1996) Molecular determinants of acute single-cell lysis by human immunodeficiency virus type 1. *J. Virol.* **70**, 1340–1354.
81. Dimitrov, D.S., Broder, C.C., Berger, E.A., and Blumenthal, R. (1993) Calcium ions are required for cell fusion mediated by the CD4-human immunodeficiency virus type 1 envelope glycoprotein interaction. *J. Virol.* **67**, 1647–1652.
82. Rucker, J., Doranz, B.J., Edinger, A.L., Long, D., Berson, J.F., and Doms, R.W. (1997) Cell-cell fusion assay to study role of chemokine receptors in human immunodeficiency virus type 1 entry. *Methods Enzymol.* **288**, 118–133.
83. Shinn-Thomas, J.H., Scranton, V.L., and Mohler, W.A. (2008) Quantitative assays for cell fusion. *Methods Mol. Biol.* **475**, 347–361.
84. Lopez-Balderas, N., Huerta, L., Villarreal, C., Rivera-Toledo, E., Sandoval, G., Larralde, C., and Lamoyi, E. (2007) In vitro cell fusion between CD4(+) and HIV-1 Env(+) T cells generates a diversity of syncytia varying in total number, size and cellular content. *Virus Res.* **123**, 138–146; 84a. Huerta, L., Lamoyi, E., Báez-Saldaña, A., and

Larralde, C. (2002) Human immunodeficiency virus envelope-dependent cell-cell fusion: a quantitative fluorescence cytometric assay. *Cytometry* **47**, 100–106.

85. Wu, P. and Brand, L. (1994) Resonance energy transfer: methods and applications. *Anal. Biochem*. **218**, 1–13.
86. Esser, U., Speck, R.F., Deen, K.C., Atchison, R.E., Sweet, R., and Goldsmith, M.A. (2000) Molecular function of the CD4 D1 domain in coreceptor-mediated entry by HIV type 1. *AIDS Res. Hum. Retroviruses* **16**, 1845–1854.
87. Huerta, L., Lopez-Balderas, N., Larralde, C., and Lamoyi, E. (2006) Discriminating in vitro cell fusion from cell aggregation by flow cytometry combined with fluorescence resonance energy transfer. *J. Virol. Methods* **138**, 17–23.
88. Furuta, R.A., Wild, C.T., Weng, Y., and Weiss, C.D. (1998) Capture of an early fusion-active conformation of HIV-1 gp41. *Nat. Struct. Biol*. **5**, 276–279.
89. Hong, Y.L., Wu, L.H., Cui, M., McMaster, G., Hunt, S.W., 3rd, and Chung, F.Z. (1999) New reporter cell lines to study macrophage-tropic HIV envelope protein-mediated cell-cell fusion. *AIDS Res. Hum. Retroviruses* **15**, 1667–1672.
90. Sakamoto, T., Ushijima, H., Okitsu, S., Suzuki, E., Sakai, K., Morikawa, S., and Muller, W.E. (2003) Establishment of an HIV cell-cell fusion assay by using two genetically modified HeLa cell lines and reporter gene. *J. Virol. Methods* **114**, 159–166.
91. Wild, C.T., Shugars, D.C., Greenwell, T.K., McDanal, C.B., and Matthews, T.J. (1994) Peptides corresponding to a predictive alpha-helical domain of human immunodeficiency virus type 1 gp41 are potent inhibitors of virus infection. *Proc. Natl. Acad. Sci. U. S. A*. **91**, 9770–9774.
92. Liu, S., Lu, H., Niu, J., Xu, Y., Wu, S., and Jiang, S. (2005) Different from the HIV fusion inhibitor C34, the anti-HIV drug Fuzeon (T-20) inhibits HIV-1 entry by targeting multiple sites in gp41 and gp120. *J. Biol. Chem*. **280**, 11259–11273.
93. Pauling, L., Campbell, D.H., and Pressman, D. (1943) The nature of forces between antigen and antibody and of the precipitation reaction. *Physiol. Rev*. **23**, 203–219.
94. Salzman, G.C. (2007) Light scatter: detection and usage. In *Current Protocols in Cytometry*. Robinson, J.P., Darzynkiewicz, Z., Hoffman, R., Nolan, J.P., Orfao, A., Rabinovitch, P.S., and Watkins, S., Eds. John Wiley & Sons, New York. Suppl. 9. pp. 1.13.1–1.13.8.
95. Pleskoff, O., Seman, M., and Alizon, M. (1995) Amphotericin B derivative blocks human immunodeficiency virus type 1 entry after CD4 binding: effect on virus-cell fusion but not on cell-cell fusion. *J. Virol*. **69**, 570–574.
96. Hioe, C.E., Bastiani, L., Hildreth, J.E., and Zolla-Pazner, S. (1998) Role of cellular adhesion molecules in HIV type 1 infection and their impact on virus neutralization. *AIDS Res. Hum. Retroviruses* **14(Suppl 3),** S247–254.
97. Allaway, G.P., Ryder, A.M., Beaudry, G.A., and Maddon, P.J. (1993) Synergistic inhibition of HIV-1 envelope-mediated cell fusion by CD4-based molecules in combination with antibodies to gp120 or gp41. *AIDS Res. Hum. Retroviruses* **9**, 581–587.
98. McKeating, J.A., Zhang, Y.J., Arnold, C., Frederiksson, R., Fenyo, E.M., and Balfe, P. (1996) Chimeric viruses expressing primary envelope glycoproteins of human immunodeficiency virus type I show increased sensitivity to neutralization by human sera. *Virology* **220**, 450–460.
99. Posner, M.R., Hideshima, T., Cannon, T., Mukherjee, M., Mayer, K.H., and Byrn, R.A. (1991) An IgG human monoclonal antibody that reacts with HIV-1/GP120, inhibits virus binding to cells, and neutralizes infection. *J. Immunol*. **146**, 4325–4332.
100. Trkola, A., Ketas, T., Kewalramani, V.N., Endorf, F., Binley, J.M., Katinger, H., Robinson, J., Littman, D.R., and Moore, J.P. (1998) Neutralization sensitivity of human immunodeficiency virus type 1 primary isolates to antibodies and CD4-based reagents is independent of coreceptor usage. *J. Virol*. **72**, 1876–1885.
101. Zwick, M.B., Wang, M., Poignard, P., Stiegler, G., Katinger, H., Burton, D.R., and Parren, P.W. (2001) Neutralization synergy of human immunodeficiency virus type 1 primary isolates by cocktails of broadly neutralizing antibodies. *J. Virol*. **75**, 12198–12208.
102. Pantaleo, G., Demarest, J.F., Vaccarezza, M., Graziosi, C., Bansal, G.P., Koenig, S., and Fauci, A.S. (1995) Effect of anti-V3 antibodies on cell-free and cell-to-cell human immunodeficiency virus transmission. *Eur. J. Immunol*. **25**, 226–231.
103. Huerta, L., Gomez-Icazbalceta, G., Soto-Ramirez, L., Viveros-Rogel, M., Rodriguez, R., Fuentes, L., Lamoyi, E., and Larralde, C. (2005) Human immunodeficiency virus 1 (HIV-1) envelope-dependent cell-cell fusion modulation by HIV-positive sera is related to disease progression. *J. Gen. Virol*. **86**, 1961–1966.
